# Photon-counting detector CTA to assess intracranial stents and flow diverters: an *in vivo* study with ultrahigh-resolution spectral reconstructions

**DOI:** 10.1186/s41747-025-00550-9

**Published:** 2025-01-29

**Authors:** Frederic De Beukelaer, Sophie De Beukelaer, Laura L. Wuyts, Omid Nikoubashman, Mohammed El Halal, Iliana Kantzeli, Martin Wiesmann, Hani Ridwan, Charlotte S. Weyland

**Affiliations:** 1 Department of Neuroradiology, University hospital RWTH Aachen, Aachen, Germany; 2 Department of Neurology, Inselspital, University hospital Bern, Bern, Switzerland; 3 Department of Radiology, AZ Sint-Lucas, Ghent, Belgium

**Keywords:** Artifacts, Cerebral arterial diseases, Computed tomography angiography, Intracranial aneurysm, Stents

## Abstract

**Background:**

To define optimal parameters for the evaluation of vessel visibility in intracranial stents (ICS) and flow diverters (FD) using photon-counting detector computed tomography angiography (PCD-CTA) with spectral reconstructions.

**Methods:**

We retrospectively analyzed consecutive patients with implanted ICS or FD, who received a PCD-CTA between April 2023 and March 2024. Polyenergetic, virtual monoenergetic, pure lumen, and iodine reconstructions with different keV levels (40, 60, and 80) and reconstruction kernels (body vascular [Bv]48, Bv56, Bv64, Bv72, and Bv76) were evaluated by two radiologists with regions of interests and Likert scales. Reconstructions were compared in descriptive analysis.

**Results:**

In total, twelve patients with nine FDs and six ICSs were analyzed. In terms of quantitative image quality, sharper kernels as Bv64 and Bv72 yielded increased image noise and decreased signal-to-noise and contrast-to-noise ratios compared to the smoothest kernel Bv48 (*p* = 0.001). Among the different keV levels and kernels, readers selected the 40 keV level (*p* = 0.001) and sharper kernels (in the majority of cases Bv72) as the best to visualize the in-stent vessel lumen. Assessing the different spectral reconstructions virtual monoenergetic and iodine reconstructions proved to be best to evaluate in-stent vessel lumen (*p* = 0.001).

**Conclusion:**

PCD-CTA and spectral reconstructions with sharper reconstruction kernels and a low keV level of 40 seem to be beneficial to achieve optimal image quality for the evaluation of ICS and FD. Iodine and virtual monoenergetic reconstructions were superior to pure lumen and polyenergetic reconstructions to evaluate in-stent vessel lumen.

**Relevance statement:**

PCD-CTA offers the opportunity to reduce the need for invasive angiography serving as follow-up examination after intracranial stent (ICS) or flow diverter (FD) implantation.

**Key Points:**

- Neuroimaging of intracranial vessels with implanted stents and flow diverters is limited by artifacts.
- Twelve patients with nine flow diverters and six intracranial stents underwent photon-counting detector computed tomography angiography (PCD-CTA).
- In-stent vessel lumen visibility improved using sharp reconstruction kernels and a low keV level.
- Virtual monoenergetic and iodine reconstructions were best to evaluate in-stent vessel lumen.

**Graphical Abstract:**

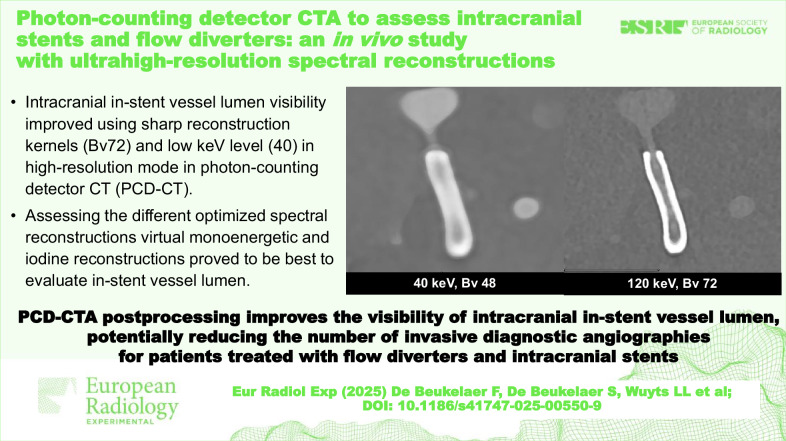

## Background

Photon-counting detector (PCD) computed tomography (CT) offers high spatial resolution and various spectral reconstruction possibilities to improve neurovascular imaging where energy-integrating-detector CT is limited [1–3].

Phantom studies for PCD-CT reported promising results for stent assessment with reduced artifacts [4]. In comparison to energy-integrating detector CT, two studies describing a total of 17 patients found higher iodine contrast attenuation (20.8%) with virtual monoenergetic imaging (VMI) reconstructions and less beam-hardening in regions with extensive bone involvement (C2 segment of the carotid artery) [5, 6]. *In vivo* studies comparing different reconstruction kernels identified Bv48 in the cerebrovascular angiography module as being optimal for assessing intracranial aneurysms [7].

Intracranial stents (ICS) and flow diverters (FD) are composed of different materials with high atomic numbers that all cause blooming artifacts to a different extent but, ultimately, artificial lumen narrowing in CT and magnetic resonance imaging. Strategies to reduce blooming artifacts in CT angiography (CTA) were higher-resolution scans and dual-energy scans with subtraction and deep learning postprocessing approaches with partial reduction of artifacts [8, 9]. Thus, digital subtraction angiography is still the standard of care method to assess stent deformation, in stent thrombosis or stenosis due to its high resolution and its insusceptibility to metal artifacts [10]. Cardiovascular CTA have identified higher kernels as better to evaluate coronary arteries. The vessel lumen of coronary arteries after stent implantation was best evaluated at sharper reconstruction kernels; body vascular (Bv) 60 and 72 [11]. Studies reporting on imaging postprocessing in PCD-CT involving spectral reconstructions are scarce in neurovascular imaging. *In vitro* studies suggest that for monoenergetic reconstructions the optimal energy threshold to identify iodine is between 40 and 60 keV [12].

ICS and FD have become widely used in the past years to treat intracranial stenosis and wide-necked aneurysms. PCD-CTA using different keV levels and reconstruction kernels might offer the possibility to assess patients with implanted ICS or FD using noninvasive imaging as compared to digital subtraction angiography for patient follow-up.

We aimed to assess the postprocessing possibilities of PCD-CTA to find the best reconstruction kernels and keV levels to assess lumen visibility and artifacts of different ICS and FD.

## Methods

This study was approved by the local ethics committee (see “Declarations”). The study fulfills the STROBE criteria, and the study was conducted following the International Declaration of Helsinki [13].

This is a single-center retrospective analysis of consecutive patients who were treated for intracranial atherosclerotic disease with symptomatic, high-grade stenosis or wide-necked aneurysms using ICS or FD between April 2023 and May 2024.

### PCD-CT protocol, postprocessing, and image analysis

Patients underwent CTA centered on the implanted device acquired on a PCD-CT scanner (NAEOTOM Alpha, Siemens Healthineers, Erlangen, Germany) operated in ultrahigh-resolution mode. The protocol was the following:

Patients underwent a CTA centered on the implanted device acquired on a clinical first-generation PCD-CT scanner (NAEOTOM Alpha, Siemens Healthineers, Erlangen, Germany) operated in ultrahigh-resolution mode, resulting in a reconstructed slice thickness of 0.2 mm and slice increment of 0.1 mm. The following acquisition parameters were used: tube voltage 140 or 120 kvp, pitch 0.65, rotation time 0.5 s. CTA was performed after the administration of 80 mL iodinated contrast material (Ultravist-370 (generic name, iopromide; Bayer Healthcare, Berlin, Germany)), injected through a 20-gauge intravenous antecubital vein catheter using a power injector. The flow rate was 4 mL/s. Opacification of the common carotid artery was monitored using a bolus tracking technique. The start time of data acquisition was determined with a fixed delay of five seconds after the attenuation threshold was reached.

**Fig. 1:**
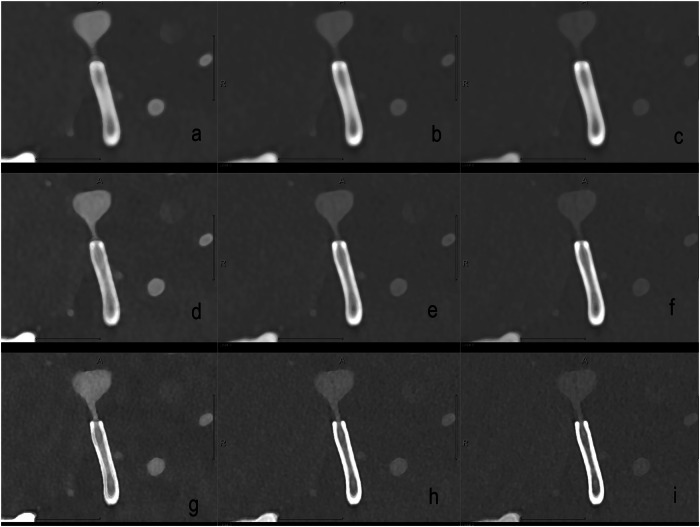
Reconstruction protocol for VMI with three different kernels and three keV levels. In the three rows, from left to right, the three keV levels (40, 80, and 120) are listed, and in the three columns, from top to bottom, 3 (instead of 5) kernels are listed as an example: Bv 46, Bv 56, and Bv 72. In detail: **a** 40 keV, Bv 48; **b** 80 keV, Bv 48; **c** 120 keV, Bv 48; **d** 40 keV, Bv 56; **e** 80 keV, Bv 56; **f** 120 keV, Bv 56; **g** 40 keV, Bv 72; **h** 80 keV; Bv 72; **i** 120 keV, Bv 72. Bv, Body Vascular kernel; VMI, Virtual monoenergetic images

Polyenergetic (PE), virtual monoenergetic imaging (VMI), pure lumen (PL) and iodine (IOD) reconstructions with different keV levels (40, 60, and 80) were obtained and systematically assessed as visualized in Fig. 1. Postprocessing details involving spectral reconstructions were as follows: Spectral reconstructions were reconstructed in 0.4 mm slice thickness and 0.2 mm slice increment. The reconstructed matrix size was 1,024 × 1,024, and the field of view was adjusted for each patient to optimally image the in-stent vessel. For PL reconstructions only 512 × 512 matrix size was available. Iterative reconstruction (denoted “QIR” by the manufacturer) level 3 was used for all PCD-CTA images. PE reconstructions in UHR mode, as well as spectral reconstructions, were performed for IOD, PL and VMI. Five image sets were reconstructed using the following vascular kernels: Bv48, Bv56, Bv64, Bv72 and Bv76 (Bv80 instead of Bv76 for PE reconstructions). For PL and VMI reconstructions, three keV levels (40, 60, and 80) were reconstructed for each kernel.

Two radiologists with 9 years and 10 years of experience assessed all images independently on a dedicated workstation (Syngo.via, version VB10A, Siemens Healthineers). Regions of interest (ROIs) were manually placed in each reconstruction, with a total of five ROIs for each reconstruction:

- one in the proximal parent vessel;
- one in the proximal end of the ICS or FD;

- one in the middle of the ICS or FD;
- one in the air rostral to the forehead, to assess the contrast-to-noise ratio (CNR);
- and one in the superficial temporal muscle.

ROIs were copied and then pasted into the other three reconstructed images, allowing to place the ROIs at the exact same location with the exact same size on every PCD-CTA reconstruction.

The size of the ROIs was as large as possible, ensuring that only the lumen of the artery was measured, carefully avoiding the stent struts. Blooming artifacts causing artificial lumen narrowing were included in the ROI. Signal was defined as the average density in HU and noise as the standard deviation (SD) of ROI density. For the calculation of the signal-to-noise ratio (SNR) and CNR, the muscle density in the superficial temporal muscle (signal_muscle_) and the SD of the air (SD_air_) adjacent to the rostral neurocranium were measured. SNR and CNR were calculated as follows:

$${{\rm{SNR}}}={{{\rm{signal}}}}_{{{\rm{artery}}}}/{{{\rm{SD}}}}_{{{\rm{artery}}}}$$

$${{\rm{CNR}}}=({{{\rm{signal}}}}_{{{\rm{artery}}}}{{{-}}}{{{\rm{signal}}}}_{{{\rm{muscle}}}})/{{{\rm{SD}}}}_{{{\rm{air}}}}$$

The two readers assessed image quality of the in-stent lumen visibility at the proximal end of the stent and in the stent using a 5-point Likert scale, ranging from 1 (“non-diagnostic”) to 5 (“excellent”).

### Statistics

IBM SPSS Statistics software (version 28.0) was used for statistical analysis. Normal distribution for descriptive analysis was tested using the Shapiro–Wilk test. Quantitative variables were expressed as mean ± SD or median and interquartile ranges. Scores from the qualitative image quality analysis were pooled across readers. The Friedman test was used to compare the different kernels in each reconstruction (IOD, PL, PE, and VMI) with pairwise comparisons. In case of the VMI and PL reconstructions, the three keV levels were compared for each kernel before using the test. Friedman test was used to compare the Likert values of the different optimized spectral reconstructions (IOD, PL, PE, and VMI). Interobserver agreement was expressed as Cohen κ value and interpreted as follows: ≤ 0.20 as none-to-slight, 0.21–0.40 as fair, 0.41–0.60 as moderate, 0.61–0.80 as substantial, and ≥ 0.81 as almost perfect agreement. All *p*-values were corrected for multiple testing using the Bonferroni correction. Reliability of the Likert scale was tested using Cronbach α test. A 95% confidence interval was calculated and expressed for the results of all diagnostic accuracy tests. A two-tailed *p*-value lower than 0.05 was considered statistically significant.

Overall, 480 reconstructions were evaluated and 2,400 ROIs were placed. Results were visualized with ggplot2 and Likert packages within R Software (The R Project for Statistical Computing, r-project.org) [14].

## Results

Patient population characteristics are reported in Table 1. The study population included twelve patients, of which eleven were women. Mean age at the time of the PCD-CTA scan was 55.5 ± 12.7 years. The indications of the PCD-CTA included aneurysm follow-up or post interventional control.

**Table 1:** Patient characteristics

Age (years)*		55.5 ± 12.7
Sex (female/male)		11 / 1
Aneurysm location	Internal cerebral artery	5
	Medial cerebral artery	1 (13)
	Anterior cerebral artery	0
	Posterior communicating artery	1 (13)
	Vertebral artery	1 (13)
	Basilar artery	0
Stenosis location	Internal cerebral artery	1 (25)
	Medial cerebral artery	1 (25)
	Anterior cerebral artery	1 (25)
	Posterior communicating artery	0
	Vertebral artery	0
	Basilar artery	1 (25)
Flow diverter type	p48 HPC	2
	p64 HPC	6
Stent type	Acclino	3
	Credo	2
	Neuroform atlas	1
In-stent vessel diameter (millimeter)*		2.7 (± 0.67)
Dose length product (mGycm)*		146 (± 75)
Dose area product (mGycm^2^)*		13,960 (± 5,403)

Except where indicated, data are numbers of participants, with percentages in parentheses

*HPC* Hydrophilic polymer coating

* Data are means ± standard deviations

Eight patients had intracranial aneurysms at the following locations: 5/8 (62%) intracranial segments of the internal carotid artery, 1/8 (13%) V4-segment of the right vertebral artery, 1/8 (13%) posterior communicating artery. Seven of eight (87%) aneurysms were treated with a total of nine FD (two p48 HPC and seven p64 MW HPC, Phenox®, Bochum, Germany). One of eight (13%) aneurysms of the distal M1 segment of the right middle cerebral artery was treated with stent-assisted coiling (Acclinoflex®, Acandis GmbH, Pforzheim, Germany). Mean vessel diameter was 2.7 ± 0.65 mm.

Four stenosis of intracranial vessels were treated with a total of four stents (two CREDO® and one Acclino® (Acandis GmbH, Pforzheim, Germany), one Neuroform Atlas® (Stryker Neurovascular, Fremont, California, USA) at following locations: 1 (25%) basilar artery, 1 (25%) medial cerebral artery, 1 (25% anterior cerebral artery), 1 (25%) internal carotid artery).

**Fig. 2:**
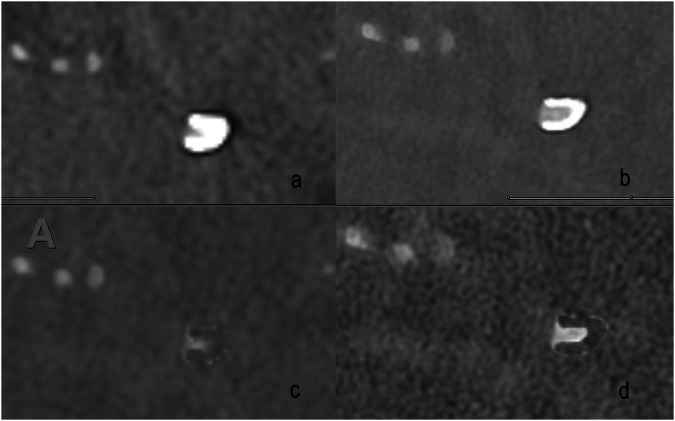
Illustrations of optimized imaging settings on visualization. **a**, **b** The distal portion of a flow diverter in the medial M1 segment of the right middle cerebral artery. **a** Ultrahigh-resolution reconstruction with Bv56 (0.2 mm); **b** Virtual monoenergetic reconstruction with Bv 72 and 40 keV; **c**, **d** Iodine reconstructions of the same vessel segment with Bv 56 (**c**) and Bv 72 (**d**). Bv, Body vascular kernel

**Fig. 3:**
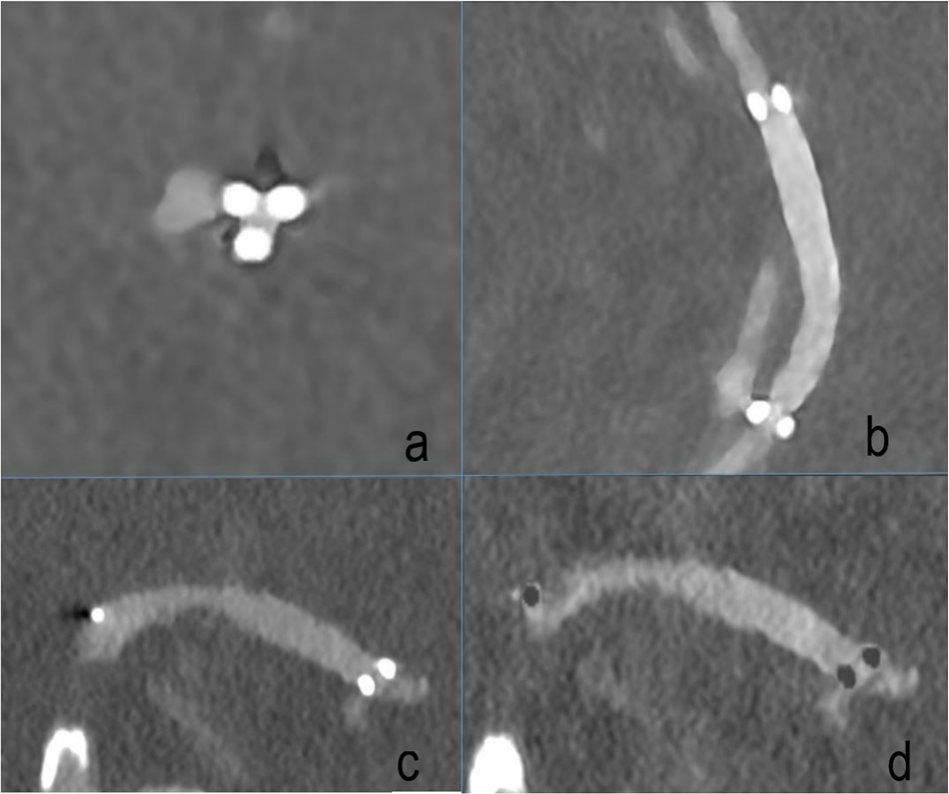
Limitations of photon-counting computed tomography angiography to overcome device-related artifacts. **a** Orthograde reconstruction of a stent at the level of the distal stent marking in the A3 segment of the left anterior cerebral artery. **b** Longitudinal section through the stent in the said vessel segment. **c** Virtual monoenergetic reconstruction (Bv72, 40 keV) and (**d**) Iodine reconstruction (Bv 72) in coronal orientation of a stent in the left middle cerebral artery. The distal stent markers at the left edge of the image are located at the transition to the proximal M2 segment. Bv, Body vascular kernel

Exemplary visualizations of in-stent-vessel lumen of PCD-CTA are provided in Fig. 2 comprising spectral reconstructions. The readers noted stent markers as a major limitation for in-stent vessel lumen visibility. Figure 3 illustrates the limitations of PCD-CTA in overcoming blooming artifacts.

### Quantitative analysis

Detailed information concerning the individual SNR/CNR values for the various reconstructions are reported in the electronic supplemental data.

For VMI reconstructions, an incremental decrease of SNR and CNR was found across the range of kernel and keV levels. The lowest keV level (40) provided the highest SNRs and CNRs, mostly with significant differences compared to the highest keV level (80). As an example, SNR values (mean ± SD) for the Bv48 kernel were higher at 40 keV than at 80 keV in the intracranial parent vessel (40 keV, 27.5 ± 11.7 *versus* 80 keV, 18.1 ± 7.5, *p* = 0.010), at the proximal ICS/FD end (46.9 ± 12.8 *versus* 4.8 ± 3.1, respectively, *p* = 0.001), and in-stent vessel lumen (21.7 ± 11.3 *versus* 9.8 ± 8.1, respectively, *p* = 0.009). Transitioning from the smoothest (Bv48) to the sharpest (Bv76) kernel, the smoothest kernel provided the highest SNRs and CNRs. As an example, SNR values for Bv48 and Bv76 kernels for 40 keV were 27.5 ± 11.7 *versus* 12.2 ± 4.7 in the intracranial parent vessel (*p* = 0.008), 46.9 ± 12.8 *versus* 7.1 ± 3.5 at the proximal ICS/FD end (*p* = 0.001), and 21.7 ± 11 *versus* 10.9 ± 4.7 for the in-stent vessel lumen (*p* = 0.001).

For pure lumen (PL) reconstructions, an incremental decrease of SNRs and CNRs was found across the range of kernels and keV levels. This reconstruction was most affected by artifacts of the implanted devices, especially stent markers. As an example, SNR values for the Bv48 and Bv76 kernels for keV 40 were 39.5 ± 25.7 *versus* 14.9 ± 3.2, respectively, in the intracranial parent vessel (*p* = 0.001). Significant differences of SNR and CNR values for the in-stent vessel lumen could not be detected across different kernels and keV levels.

For IOD and PE reconstructions, smoother kernels consistently provided the highest SNRs and CNRs in the parent vessel and in the in-stent-vessel lumen.

### Qualitative analysis

**Fig. 4:**
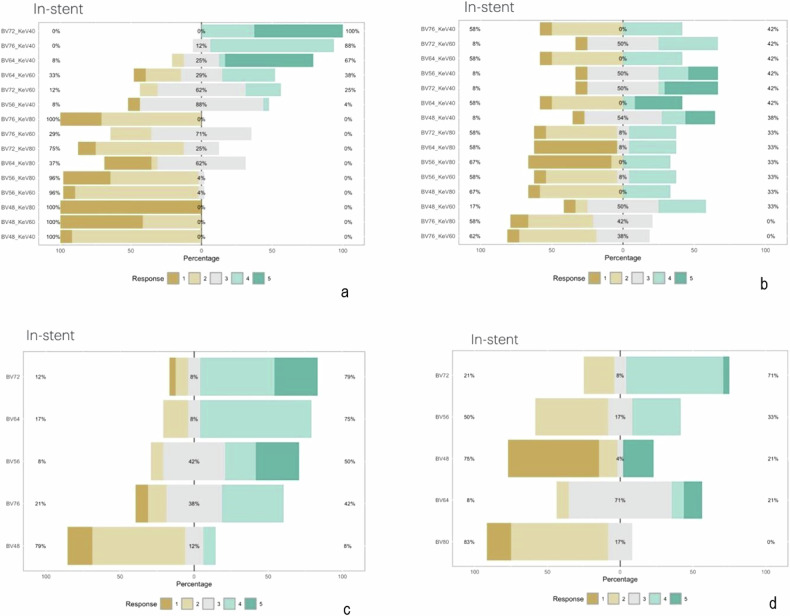
Image quality scores of the in-stent vessel segment of spectral reconstructions—VMI (**a**), PL (**b**), and IOD (**c**)—with different kernels (Bv48, Bv56, Bv64, Bv72, Bv76) and keV levels (40, 80, 120) as well as PE reconstructions (**d**) with the same kernels (except for Bv80 that was used instead of Bv76). Stacked bar charts show pooled percentages of two raters, at three different sites: proximal vessel, proximal stent, and in-stent. Image quality of scores: 5 = excellent; 4 = good; 3 = acceptable; 2 = barely satisfactory; 1 = unacceptable. Bv, Body vascular; IOD, Iodine; PE, Polyenergetic reconstructions; PL, Pure lumen; VMI, Virtual monoenergetic imaging

Likert values of spectral reconstructions and PE angiography are displayed in Fig. 4. Substantial agreement (κ = 0.72) was found between the two readers for qualitative ratings using the 5-point Likert-type scale. The internal consistency of the test, measured by Cronbach α, was excellent (0.89). For VMI, reconstructions with 40 keV achieved the highest score (*p* < 0.001). For the parent vessel images reconstructed with Bv56 kernel achieved the highest scores (*p* < 0.001).

For the in-stent vessel lumen, Bv64, Bv72, and Bv76 achieved higher scores than Bv56 and Bv48. Out of the three kernels, the two readers of the study favored the Bv72 kernel for the visualization of in-stent vessel lumen. For PL reconstructions, images reconstructed with the 40 keV achieved the highest scores, however, for the parent vessel as well as for the in-stent vessel lumen, there was no preferred reconstruction. For IOD reconstructions, images with the Bv56 kernel achieved higher assessment scores for the parent vessel. For evaluation of the in-stent vessel lumen, Bv72 achieved higher scores than Bv56 and Bv48. Out of the three kernels, the two readers of the study favored the Bv72 kernel. For PE reconstructions, the parent vessel images reconstructed with the kernels Bv48 and Bv56 kernels achieved the highest score. However, imaging with Bv48, Bv56, and Bv64 kernels was comparable. Out of the three kernels, the two readers of the study favored the Bv56 kernel for the visualization of the parent vessel. For the in-stent vessel lumen, the Bv64 and Bv72 kernels achieved higher scores than Bv80, Bv56 and Bv48 kernels. However, only the pairwise comparisons of Bv64 and Bv72 kernels with the Bv80 kernel were significantly different with lower quality of the Bv80 kernel *(*Bv64 *versus* Bv80*, p* = 0.008 and Bv72 *versus* Bv80, *p* < 0.001*)*. When reviewing the PCD-CTA images in consensus reading after blinded assessment, the Bv72 kernel was selected as the most suitable for the evaluation of in-stent vessel lumen. When assessing the best spectral reconstructions to evaluate in-stent vessel lumen, pairwise comparisons identified virtual monoenergetic images at 40 keV and iodine reconstructions as best to evaluate in-stent vessel lumen (*p* = 0.001).

## Discussion

The present study evaluates the objective and subjective image quality of PCD-CTA for intracranial vessels after ICS or FD implantation using different levels of kernel sharpness and keV. Our results highlight the significance of sharper kernels in enhancing the in-stent vessel lumen and reduce artificial lumen narrowing. The use of the Bv72 kernel across spectral reconstructions and, additionally, a low keV level (40 keV) for virtual monoenergetic reconstructions significantly increased vessel visibility.

Studies on kernel optimization for in-stent vessel lumen of intracranial arteries have been limited to phantom studies. Based on their objective and subjective image quality results, the authors recommended the use of Bv60 for spectral PCD-CTA [4]. This study represents a systematic approach for neurovascular imaging applications across different kernel reconstructions and spectral reconstructions using PCD-CTA.

Our quantitative analysis showed that image noise increases by utilizing sharper kernels, resulting in a continuous decrease in SNR and CNR, even for a quantum iterative reconstruction level of three. Our qualitative image analysis demonstrated a clear preference of the readers for sharper kernels. A similar observation was published recently: qualitative assessment differed from the quantitative assessment [7]. We agree with the authors’ opinion that for a comprehensive evaluation of image quality, both qualitative and quantitative assessments should be considered, as they provide complementary information. While SNR and CNR allow a reproducible, quantitative evaluation for assessing image quality, qualitative evaluation allows to integrate several features simultaneously that may be difficult to quantify. The Bv64 and Bv72 kernels both delivered superior image quality at qualitative assessment. However, the observers favored the Bv72 kernel (at a keV level of 40) for evaluating the in-stent vessel lumen.

While digital subtraction angiography remains the standard of care for the evaluation of intracranial aneurysms, PCD-CTA has the potential to offer less invasive imaging for ICS and FD evaluation. Our preliminary results may provide a valuable reference to exploit the full potential of the ultrahigh-resolution mode and spectral reconstructions of PCD-CTA for neurovascular imaging.

This study has limitations. First, we included a relatively small number (*n* = 12) of patients from a single center, which limits the generalizability of results, and implanted FD and ICS were of limited variability. Although the different stents showed varying artifact degrees, the in-stent vessel lumen was consistently better visualized across all reconstructions in the selected kernel and keV. Second, only one type of kernel (Bv) was evaluated. Third, ROIs were placed manually by a single observer, which could introduce the risk of measurement bias.

In conclusion, the evaluation of intracranial vessels after ICS or FD implantation with PCD-CTA can provide optimal image quality by using spectral reconstruction, sharper kernels, and a keV level as low as 40. IOD and VMI were superior to PL and PE reconstructions to evaluate in-stent vessel lumen. Provided these study results can be reproduced in larger patient series, PCD-CTA could offer the opportunity to reduce the need for invasive angiography as follow-up after intracranial stent or flow diverter implantation.

## Supplementary information

**Additional file 1:** **Table S1a.** Polenergetic Reconstructions: Quantitative image quality measurements of the PCD-CT. **Table S2.** Pairwise comparison for SNR and CNR in proximal vessel, proximal stented vessel and stented vessel for polyenergetic reconstructions. **a** Pairwise comparison for SNR in proximal vessel. **b** Pairwise comparison for CNR in proximal vessel. **c** Pairwise comparison for SNR in proximal stented vessel. **d** Pairwise comparison for CNR in proximal stented vessel. **e** Pairwise comparison for SNR in stented vessel. **f** Pairwise comparison for CNR in stented vessel. **Table S3.** Iodine Reconstructions: Quantitative image quality measurements of the PCD-CT. **Table S4a.** Pairwise comparison for SNR and CNR in proximal vessel, proximal stented vessel and stented vessel for iodine reconstructions. **a** Pairwise comparison for SNR in proximal vessel. **b** Pairwise comparison for CNR in proximal vessel. **c** Pairwise comparison for SNR in proximal stented vessel. **d** Pairwise comparison for CNR in proximal stented vessel. **e** Pairwise comparison for SNR in stented vessel. **f** Pairwise comparison for CNR in stented vessel. **Table S5.** Virtual monoenergetic reconstructions for Bv56 Kernel. Quantitative image quality measurements of the PCD-CT. **Table S6a.** Pairwise comparison for SNR in proximal vessel (PV), proximal stented (PS) vessel and stented vessel (s) for Bv56. **b** Pairwise comparison for CNR in proximal vessel (PV), proximal stented (PS) vessel and stented vessel (s) for Bv56. **Table S7.** Virtual monoenergetic reconstructions for keV level 40: Quantitative image quality measurements of the PCD-CT. **Table S8a.** Pairwise comparison for SNR in proximal vessel. **b** Pairwise comparison for CNR in proximal vessel. **c** Pairwise comparison for SNR in proximal stented vessel. **d** Pairwise comparison for CNR in proximal stented vessel. **e** Pairwise comparison for SNR in stented vessel. **f** Pairwise comparison for CNR in stented vessel. **Table 9.** Pure lumen reconstructions for Bv64 Kernel. Quantitative image quality measurements of the PCD-CT. **Table 10a.** Pairwise comparison for SNR in proximal vessel (PV), proximal stented (PS) vessel and stented vessel (s) for Bv64. **b** Pairwise comparison for CNR in proximal vessel (PV), proximal stented (PS) vessel and stented vessel (s) for Bv64. **Table 11.** Pure Lumen reconstructions for keV level of 40: Quantitative image quality measurements of the PCD-CT. **Table 12a.** Pairwise comparison for SNR in proximal vessel. **b** Pairwise comparison for CNR in proximal vessel.

## Data Availability

Data are available from the corresponding author upon reasonable request.

## Abbreviations

Bv: Body vascular (kernel)
CNR: Contrast-to-noise ratio
CT: Computed tomography
FD: Flow diverter
ICS: Intracranial stent
IOD: Iodine reconstruction
PCD: Photon-counting detector
PE: Polyenergetic
PL: Pure lumen
ROI: Region of interest
SD: Standard deviation
SNR: Signal-to-noise ratio
VMI: Virtual monoenergetic imaging

## Materials availability

Materials are available from the corresponding author upon reasonable request.
