# Supplementary material for: Photon-counting detector CTA to assess intracranial stents and flow diverters: an in vivo study with ultrahigh-resolution spectral reconstructions
Source: Eur Radiol Exp. 2025 Jan 29;9:10. doi: 10.1186/s41747-025-00550-9 (PMC11780015; doi:10.1186/s41747-025-00550-9)
Supplement: Supplementary file 1 — Additional file 1: Table S1a. Polenergetic Reconstructions: Quantitative image quality measurements of the PCD-CT. Table S2. Pairwise comparison for SNR and CNR in proximal vessel, proximal stented vessel and stented vessel for polyenergetic reconstructions. a Pairwise comparison for SNR in proximal vessel. b Pairwise comparison for CNR in proximal vessel. c Pairwise comparison for SNR in proximal stented vessel. d Pairwise comparison for CNR in proximal stented vessel. e Pairwise comparison for SNR in stented vessel. f Pairwise comparison for CNR in stented vessel. Table S3. Iodine Reconstructions: Quantitative image quality measurements of the PCD-CT. Table S4a. Pairwise comparison for SNR and CNR in proximal vessel, proximal stented vessel and stented vessel for iodine reconstructions. a Pairwise comparison for SNR in proximal vessel. b Pairwise comparison for CNR in proximal vessel. c Pairwise comparison for SNR in proximal stented vessel. d Pairwise comparison for CNR in proximal stented vessel. e Pairwise comparison for SNR in stented vessel. f Pairwise comparison for CNR in stented vessel. Table S5. Virtual monoenergetic reconstructions for Bv56 Kernel. Quantitative image quality measurements of the PCD-CT. Table S6a. Pairwise comparison for SNR in proximal vessel (PV), proximal stented (PS) vessel and stented vessel (s) for Bv56. b Pairwise comparison for CNR in proximal vessel (PV), proximal stented (PS) vessel and stented vessel (s) for Bv56. Table S7. Virtual monoenergetic reconstructions for keV level 40: Quantitative image quality measurements of the PCD-CT. Table S8a. Pairwise comparison for SNR in proximal vessel. b Pairwise comparison for CNR in proximal vessel. c Pairwise comparison for SNR in proximal stented vessel. d Pairwise comparison for CNR in proximal stented vessel. e Pairwise comparison for SNR in stented vessel. f Pairwise comparison for CNR in stented vessel. Table 9. Pure lumen reconstructions for Bv64 Kernel. Quantitative image quality [file 41747_2025_550_MOESM1_ESM.pdf]

Photon-counting detector CTA to assess intracranial stents and flow diverters: an in vivo study with ultrahigh-resolution spectral reconstructions

ELECTRONIC SUPPLEMENTARY MATERIAL

Table S1a. Polenergetic Reconstructions: Quantitative image quality measurements of the PCD-CT.

	Bv48	Bv56	Bv64	Bv72	Bv80	P
Lumen of proximal artery						
SNR	24.7	17.1	10.7	8.3	3.1	<0.01
CNR	26.2	15.9	10.6	9.2	4.7	<0.01
Lumen of proximal stent/flowdiverter end						
SNR	10.4	10.3	8.1	6.1	3.2	<0.01
CNR	30.9	16.3	12.5	10.5	5.3	<0.01
Lumen of stent/flowdiverter						
SNR	18.9	14.1	9.6	7.5	4.2	<0.01
CNR	25.3	16.4	11.4	10.1	5.1	<0.01

All values are reported are means, medians are marked with a * (Bv: body vascular, CNR: Contrast-to-noise Ratio, IQR: Interquartile range, SD: standard deviation, SNR: signal- to-noise ratio)

Table S2 Pairwise comparison for SNR and CNR in proximal vessel, proximal stented vessel and stented vessel for polyenergetic reconstructions

Table 2a Pairwise comparison for SNR in proximal vessel

	Bv80-Bv72	BV80-BV64	Bv80 - Bv56	Bv80 - Bv48	Bv72 - Bv64	Bv72 - Bv 56	Bv72 - Bv48	Bv64 - Bv56	Bv64 - Bv48	Bv56 - Bv48
P	.09	<0.01	<0.01	<0.01	.2	<0.01	<0.01	.07	.02	.2
P*	.93	.03	<0.01	<0.01	1	.02	<0.01	.71	.02	1

Table 2b Pairwise comparison for CNR in proximal vessel

	Bv80-Bv72	BV80-BV64	Bv80 - Bv56	Bv80 - Bv48	Bv72 - Bv64	Bv72 - Bv 56	Bv72 - Bv48	Bv64 - Bv56	Bv64 - Bv48	Bv56 - Bv48
P	.07	<0.01	<0.01	<0.01	.34	.01	<0.01	.12	<0.01	.07
P*	.71	.07	<0.01	<0.01	1	.14	<0.01	1	<0.01	.71

Table 2c Pairwise comparison for SNR in proximal stented vessel

	Bv80-Bv72	BV80-BV64	Bv80 - Bv56	Bv80 - Bv48	Bv72 - Bv64	Bv72 - Bv 56	Bv72 - Bv48	Bv64 - Bv56	Bv64 - Bv48	Bv56 - Bv48
P	.04	<0.01	<0.01	<0.01	.35	.03	.02	.23	.18	.89
P*	.43	.03	<0.01	<0.01	.1	.31	.22	1	1	1

Table 2d Pairwise comparison for CNR in proximal stented vessel

	Bv80-Bv72	BV80-BV64	Bv80 - Bv56	Bv80 - Bv48	Bv72 - Bv64	Bv72 - Bv 56	Bv72 - Bv48	Bv64 - Bv56	Bv64 - Bv48	Bv56 - Bv48
P	.06	.01	<0.01	<0.01	.5	.04	<0.01	.18	<0.01	.06
P*	.59	.11	<0.01	<0.01	1	.43	<0.01	1	.01	.59

Table 2e Pairwise comparison for SNR in stented vessel

	Bv80-Bv72	BV80-BV64	Bv80 - Bv56	Bv80 - Bv48	Bv72 - Bv64	Bv72 - Bv 56	Bv72 - Bv48	Bv64 - Bv56	Bv64 - Bv48	Bv56 - Bv48
P	.09	<0.01	<0.01	<0.01	.25	<0.01	<0.01	.05	.04	.89
P*	.93	.05	<0.01	<0.01	1	.02	.01	.53	.39	1

2f Pairwise comparison for CNR in stented vessel

	Bv80-Bv72	BV80-BV64	Bv80 - Bv56	Bv80 - Bv48	Bv72 - Bv64	Bv72 - Bv 56	Bv72 - Bv48	Bv64 - Bv56	Bv64 - Bv48	Bv56 - Bv48
P	.07	<0.01	<0.01	<0.01	.25	.02	<0.01	.25	<0.01	.07
P*	.71	.03	<0.01	<0.01	1	.2	<0.01	1	.03	.71

Note

P: p-value, P*: P-value adapted after Bonferroni-Correction. All values are reported are means, medians are marked with a * (Bv: body vascular, CNR: Contrast-to-noise Ratio, IQR: Interquartile rande, SD: standard deviation, SNR: signal- to-noise ratio)

Table S3. Iodine Reconstructions: Quantitative image quality measurements of the PCD-CT. All values are reported as means and SD (Bv: body vascular, CNR: Contrast-to-noise Ratio, IQR: Interquartile rande, SD: standard deviation, SNR: signal- to-noise ratio

	Bv48	Bv56	Bv64	Bv72	Bv76	P	
Lumen of proximal artery							
SNR	24.6	15.9	10.2	8.7	6.6	<0.01	
CNR	224.9	213.9	189.5	194.5	189.9	<0.01	
Lumen of proximal stent/flowdiverter end							
SNR	3.3	4.0	5.1	4.4	3.9	<0.01	
CNR	224.9	186.9	167.1	-5.3	187.7	<0.01	
Lumen of stent/flowdiverter							
SNR	9.2	9.1	7.6	7.1	5.7	<0.01	
CNR	220.9	198.6	200.9	191.6	188.2	<0.01	

All values are reported are means, medians are marked with a * (Bv: body vascular, CNR: Contrast-to-noise Ratio, IQR: Interquartile range, SD: standard deviation, SNR: signal- to-noise ratio)

Table S4a Pairwise comparison for SNR and CNR in proximal vessel, proximal stented vessel and stented vessel for iodine reconstructions

Pairwise comparison for SNR in proximal vessel

	Bv76-Bv72	BV76-BV64	Bv76 - Bv56	Bv76 - Bv48	Bv72 - Bv64	Bv72 - Bv 56	Bv72 - Bv48	Bv64 - Bv56	Bv64 - Bv48	Bv56 - Bv48
P	.1	<0.01	<0.01	<0.01	.19	.02	<0.01	.27	<0.01	.09
P*	1	.04	<0.01	<0.01	1	.17	<0.01	1	.06	.9

P: p-value, P*: P-value adapted after Bonferroni-Correction

4b Pairwise comparison for CNR in proximal vessel

	Bv76-Bv72	BV76-BV64	Bv76 - Bv56	Bv76 - Bv48	Bv72 - Bv64	Bv72 - Bv 56	Bv72 - Bv48	Bv64 - Bv56	Bv64 - Bv48	Bv56 - Bv48
P	.61	.16	.04	<0.01	.37	.12	<0.01	.52	<0.01	.05
P*	1	1	.39	<0.01	1	1	<0.01	1	.08	.45

4c Pairwise comparison for SNR in proximal stented vessel

	Bv76-Bv72	BV76-BV64	Bv76 - Bv56	Bv76 - Bv48	Bv72 - Bv64	Bv72 - Bv 56	Bv72 - Bv48	Bv64 - Bv56	Bv64 - Bv48	Bv56 - Bv48
P	.67	.05	.03	.01	.121	.07	.03	.79	.52	.67
P*	1	.53	.28	.1	1	.71	.28	1	1	1

4d Pairwise comparison for CNR in proximal stented vessel

	Bv76-Bv72	BV76-BV64	Bv76 - Bv56	Bv76 - Bv48	Bv72 - Bv64	Bv72 - Bv 56	Bv72 - Bv48	Bv64 - Bv56	Bv64 - Bv48	Bv56 - Bv48
P	<0.01	<0.01	<0.01	<0.01	.52	.44	.01	.89	.05	.07
P*	.07	<0.01	<0.01	<0.01	1	1	.1	1	.53	.71

4e Pairwise comparison for SNR in stented vessel

	Bv76-Bv72	BV76-BV64	Bv76 - Bv56	Bv76 - Bv48	Bv72 - Bv64	Bv72 - Bv 56	Bv72 - Bv48	Bv64 - Bv56	Bv64 - Bv48	Bv56 - Bv48
P	.09	<0.01	<0.01	<0.01	.25	<0.01	<0.01	.05	.04	.89
P*	.93	.05	<0.01	<0.01	1	.02	.01	.53	.39	1

4f Pairwise comparison for CNR in stented vessel

	Bv76-Bv72	Bv76-Bv64	Bv76 - Bv56	Bv76 - Bv48	Bv72 - Bv64	Bv72 - Bv 56	Bv72 - Bv48	Bv64 - Bv56	Bv64 - Bv48	Bv56 - Bv48
P	.07	<0.01	<0.01	<0.01	.25	.02	<0.01	.25	. <0.01	.07
P*	.71	.03	<0.01	<0.01	1	.2	<0.01	1	.03	.71

Table S5 Virtual monoenergetic reconstructions for Bv56 Kernel. Quantitative image quality measurements of the PCD-CT. All values are reported as means and SD (Bv: body vascular, CNR: Contrast-to-noise Ratio, IQR: Interquartile rande, SD: standard deviation, SNR: signal-to-noise ratio

	keV 40	keV 60	keV 80			P	
Lumen of proximal artery							
SNR	25.7	19.9	12.9			<0.01	
CNR	33.7	21.1	14.8			<0.01	
Lumen of proximal stent/flowdiverter end							
SNR	14.6	8.2*	5.9*			<0.01	
CNR	40.8	36.7*	35.4*			.05	
Lumen of stent/flowdiverter							
SNR	20.4*	14.1	10.0			<0.01	
CNR	39.1*	36.7*	21.8			<0.01	

Table S6 a Pairwise comparison for SNR in proximal vessel(PV), proximal stented (PS) vessel and stented vessel (s) for Bv56

	PV80-PV60	PV80-40	Pv60 - Pv40	PS80 - PS60	PS80 - PS40	PS60 - PS40	S80-S60	S80-S40	S60-S40
P	<0.01	<0.01	.04	.07	<0.01	.07	.07	<0.01	.014
P*	.02	<0.01	.12	.2	<0.01	.19	.19	<0.01	.04

P: p-value, P*: P-value adapted after Bonferroni-Correction

Table 6b Pairwise comparison for CNR in proximal vessel(PV), proximal stented (PS) vessel and stented vessel (s) for Bv56

	PV80-PV60	PV80-40	Pv60 - Pv40	PS80 - PS60	PS80 - PS40	PS60 - PS40	S80-S60	S80-S40	S60-S40
P	.04	<0.01	<0.01	.2	.6	.2	.07	<0.01	.22
P*	.12	<0.01	.02	.01	.04	.6	.19	<0.01	.66

P: p-value, P*: P-value adapted after Bonferroni-Correction

Table S7 Virtual monoenergetic reconstructions for keV level 40: Quantitative image quality measurements of the PCD-CT. All values are reported as means and SD (Bv: body vascular, CNR: Contrast-to-noise Ratio, IQR: Interquartile rande, SD: standard deviation, SNR: signal-to-noise ratio

	Bv48	Bv56	Bv64	Bv72	Bv76	P	
Lumen of proximal artery							
SNR	27.4	25.7	17.2*	15.6	12.9*	<0.01	
CNR	46.9	33.7	22.9	21.8	15.9	<0.01	
Lumen of proximal stent/flowdiverter end							
SNR		14.6	12.5	10.6	7.1	<0.01	
CNR	46.9	40.8	25.7	21.0*	15.3*	<0.01	
Lumen of stent/flowdiverter							
SNR	21.7	17.5*	17.3	17.6	10.9	<0.01	
CNR	49.6	32.8*	24.2	21.2*	17.6	<0.01	

All values are reported are means, medians are marked with a * (Bv: body vascular, CNR: Contrast-to-noise Ratio, IQR: Interquartile rande, SD: standard deviation, SNR: signal- to-noise ratio)

Table S8a Pairwise comparison for SNR in proximal vessel

	Bv76-Bv72	BV76-BV64	Bv76 - Bv56	Bv76 - Bv48	Bv72 - Bv64	Bv72 - Bv48	Bv72 – Bv56	Bv64 – Bv48	Bv64 – Bv56	Bv56 - Bv48
P	.16	.03	<0.01	<0.01	.44	.03	.02	.16	.12	.89
P*	1	.28	<0.01	<0.01	1	.28	.20	1	1	1

P: p-value, P*: P-value adapted after Bonferroni-Correction

Table S8b Pairwise comparison for CNR in proximal vessel

	Bv76-Bv72	BV76-BV64	Bv76 - Bv56	Bv76 - Bv48	Bv72 - Bv64	Bv72 - Bv 56	Bv72 - Bv48	Bv64 - Bv56	Bv64 - Bv48	Bv56 - Bv48
P	.034	.03	<0.01	<0.01	.89	.04	<0.01	.05	<0.01	.09
P*	.39	.28	<0.01	<0.01	1	.39	<0.01	.53	<0.01	.93

Table S8c Pairwise comparison for SNR in proximal stented vessel

	Bv76-Bv72	BV76-BV64	Bv76 - Bv56	Bv76 - Bv48	Bv72 - Bv64	Bv72 - Bv 56	Bv72 - Bv48	Bv64 - Bv56	Bv64 - Bv48	Bv56 - Bv48
P	.11	.01	<0.01	<0.01	.4	.18	<0.01	.65	.04	.12
P*	1	.12	.03	<0.01	1	1	.04	1	.45	1

Table S8d Pairwise comparison for CNR in proximal stented vessel

	Bv76-Bv72	BV76-BV64	Bv76 - Bv56	Bv76 - Bv48	Bv72 - Bv64	Bv72 - Bv 56	Bv72 - Bv48	Bv64 - Bv56	Bv64 - Bv48	Bv56 - Bv48
P	.04	.04	<0.01	<0.01	<0.01	<0.01	0.3	.3	1	.37
P*	.39	.39	<0.01	<0.01	.02	.02	.3	.3	1	1

Table S8ePairwise comparison for SNR in stented vessel

	Bv76-Bv64	BV76-BV72	Bv76 - Bv56	Bv76 - Bv48	Bv64 – Bv48	Bv64 - Bv 56	Bv72 - Bv48	Bv72 4 - Bv56	Bv64 – Bv72	Bv56 - Bv48
P	.02	<0.01	<0.01	<0.01	.44	.19	.12	.61	.44	.79
P*	.2	.02	<0.01	<0.01	1	1	1	1	1	1

Table S8f Pairwise comparison for CNR in stented vessel

	Bv76-Bv64	BV76-BV72	Bv76 - Bv56	Bv76 - Bv48	Bv64 – Bv48	Bv64 - Bv 56	Bv72 - Bv48	Bv72 - Bv56	Bv64 – Bv56	Bv56 - Bv48
P	.05	.05	<0.01	<0.01	<0.01	<0.01	1	.03	.03	.16
P*	.53	.53	<0.01	<0.01	<0.01	<0.01	1	.28	.28	1

P: p-value, P*: P-value adapted after Bonferroni-Correction

Table 9 Pure lumen reconstructions for Bv64 Kernel. Quantitative image quality measurements of the PCD-CT. All values are reported as means and SD (Bv: body vascular, CNR: Contrast-to-noise Ratio, IQR: Interquartile rande, SD: standard deviation, SNR: signal-to-noise ratio

	keV 40	keV 60	keV 80			P	
Lumen of proximal artery							
SNR	18.8	10.1	11.2			<0.01	
CNR	29.8	16.2	6.6			<0.01	
Lumen of proximal stent/flowdiverter end							
SNR	-0.3*	.47	.9			.42	
CNR	-17.7	1.05	.2			.67	
Lumen of stent/flowdiverter							
SNR	12.8	13.1*	6.9			.85	
CNR	28.5	14.3	10.1			.15	

All values are reported are means, medians are marked with a * (Bv: body vascular, CNR: Contrast-to-noise Ratio, IQR: Interquartile rande, SD: standard deviation, SNR: signal- to-noise ratio)

Table 10a Pairwise comparison for SNR in proximal vessel(PV), proximal stented (PS) vessel and stented vessel (s) for Bv64

	PV80-PV60	PV80-40	Pv60 - Pv40
P	.8	<0.01	<0.01
P*	1	<0.01	<0.01

P: p-value, P*: P-value adapted after Bonferroni-Correction

Table 10b Pairwise comparison for CNR in proximal vessel(PV), proximal stented (PS) vessel and stented vessel (s) for Bv64

	PV80-PV60	PV80-40	Pv60 - Pv40
P	.03	<0.01	.03
P*	.08	<0.01	.08

P: p-value, P*: P-value adapted after Bonferroni-Correction

Table 11 Pure Lumen reconstructions for keV level of 40: Quantitative image quality measurements of the PCD-CT. All values are reported as means and SD (Bv: body vascular, CNR: Contrast-to-noise Ratio, IQR: Interquartile rande, SD: standard deviation, SNR: signal-to-noise ratio

	Bv48	Bv56	Bv64	Bv72	Bv76	P	
Lumen of proximal artery							
SNR	39.9	20.4	17.8	22.9	14.9	<0.01	
CNR	68.3	47.9*	29.8	865	29.2	<0.01	
Lumen of proximal stent/flowdiverter end							
SNR	.28	2.9*	.3*	.6*	1.6*	.71	
CNR	-34	-99.8*	-17	-35.4	2.9	.58	
Lumen of stent/flowdiverter							
SNR	33.4	29.0	12.8	10.5	7.6	.31	
CNR	77	86.8*	28.5	34.4	13.4	03	

Table 12a Pairwise comparison for SNR in proximal vessel

	Bv76-Bv72	BV76-BV64	Bv76 - Bv48	Bv76 - Bv56	Bv72 - Bv64	Bv72 - Bv48	Bv72 – Bv56	Bv64 – Bv48	Bv64 – Bv56	Bv56 - Bv48
P	.16	.03	<0.01	<0.01	.48	.024	<0.01	.12	.05	.67
P*	1	.4	<0.01	<0.01	1	.24	.07	1	.48	1

P: p-value, P*: P-value adapted after Bonferroni-Correction

Table 12b Pairwise comparison for CNR in proximal vessel

	Bv76-Bv72	BV76-BV64	Bv76 - Bv56	Bv76 - Bv48	Bv72 - Bv64	Bv72 - Bv 56	Bv72 - Bv48	Bv64 - Bv56	Bv64 - Bv48	Bv56 - Bv48
P	.07	<0.01	<0.01	<0.01	.2	.09	<0.01	.67	.01	.03
P*	.6	.02	<0.01	<0.01	1	.89	<0.01	1	.1	.3
